# Alpha cell regulation of beta cell function

**DOI:** 10.1007/s00125-020-05196-3

**Published:** 2020-09-07

**Authors:** Tilo Moede, Ingo B. Leibiger, Per-Olof Berggren

**Affiliations:** The Rolf Luft Research Center for Diabetes and Endocrinology, Karolinska Institutet, Karolinska Sjukhuset L1:03, 17176 Stockholm, Sweden

**Keywords:** Acetylcholine, Alpha cell, Beta cell, GLP-1, Glucagon, Human, Islets, Mouse, Paracrine interaction, Review

## Abstract

The islet of Langerhans is a complex endocrine micro-organ consisting of a multitude of endocrine and non-endocrine cell types. The two most abundant and prominent endocrine cell types, the beta and the alpha cells, are essential for the maintenance of blood glucose homeostasis. While the beta cell produces insulin, the only blood glucose-lowering hormone of the body, the alpha cell releases glucagon, which elevates blood glucose. Under physiological conditions, these two cell types affect each other in a paracrine manner. While the release products of the beta cell inhibit alpha cell function, the alpha cell releases factors that are stimulatory for beta cell function and increase glucose-stimulated insulin secretion. The aim of this review is to provide a comprehensive overview of recent research into the regulation of beta cell function by alpha cells, focusing on the effect of alpha cell-secreted factors, such as glucagon and acetylcholine. The consequences of differences in islet architecture between species on the interplay between alpha and beta cells is also discussed. Finally, we give a perspective on the possibility of using an in vivo imaging approach to study the interactions between human alpha and beta cells under in vivo conditions.

**Electronic supplementary material:**

The online version of this article (10.1007/s00125-020-05196-3) contains a slideset of the figures for download, which is available to authorised users.

**Figure:**
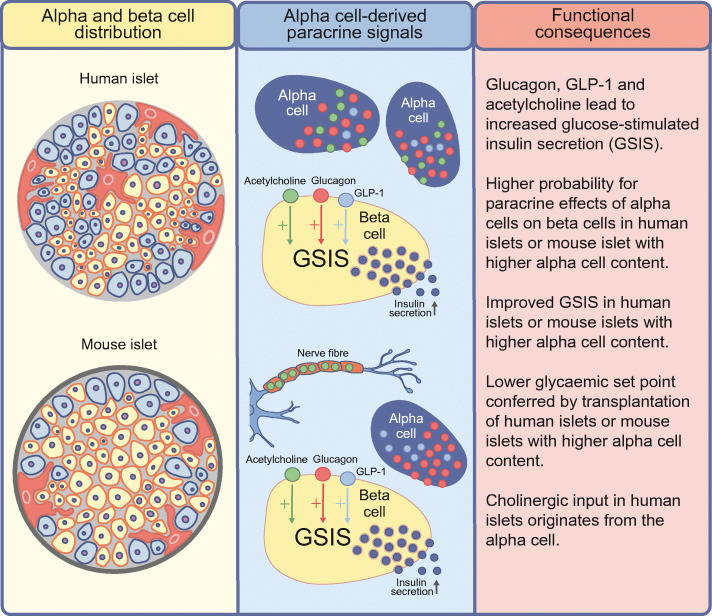
Graphical abstract

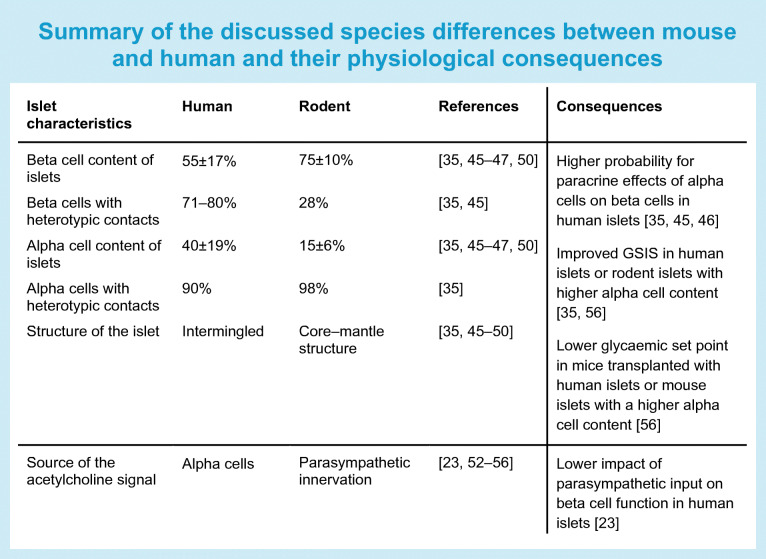



## Introduction

The islets of Langerhans, first described by Paul Langerhans in 1869, are dispersed throughout the exocrine tissue of the pancreas. Each islet is an endocrine micro-organ consisting of multiple endocrine cell types, the two most prominent and numerous of which are beta and alpha cells. Beta cells are the producers of the only blood glucose-lowering hormone in the body: insulin. Alpha cells, by contrast, produce glucagon, a hormone that has blood glucose-increasing effects. One interesting and maybe more philosophical question to consider is why these two counteracting hormones are produced in such close proximity to each other. Mathematical modelling suggests that a system in which the products of one partner, like the glucagon produced by the pancreatic alpha cell, have a stimulatory effect on its counterpart, while the other partner’s products, like the insulin secreted by the beta cell, have inhibitory effects, enables active regulation to maintain stable levels, something that is indeed mandatory for the control of blood glucose concentration [1–3]. It is therefore of interest to explore the mechanisms that are involved in the interplay between alpha and beta cells. Each islet of Langerhans also contains other endocrine cell types, such as the somatostatin-producing delta cell. Somatostatin is an inhibitor of both glucagon and insulin release and is an important regulator of glucose homeostasis [4]. However, the paracrine effects of delta cell secretion products are beyond the scope of this review. Beta cell-released factors, including, among others, insulin, Zn^2+^, ATP and γ-aminobutyric acid (GABA), have an inhibitory effect on glucagon secretion by alpha cells [5]. The focus of this review is the role of factors originating from the alpha cell in the regulation of beta cell activity and, by extension, the regulation of glucose homeostasis.

## Beta cell stimulus–secretion coupling and potential interaction points with paracrine factors secreted from the alpha cell

**Fig. 1:**
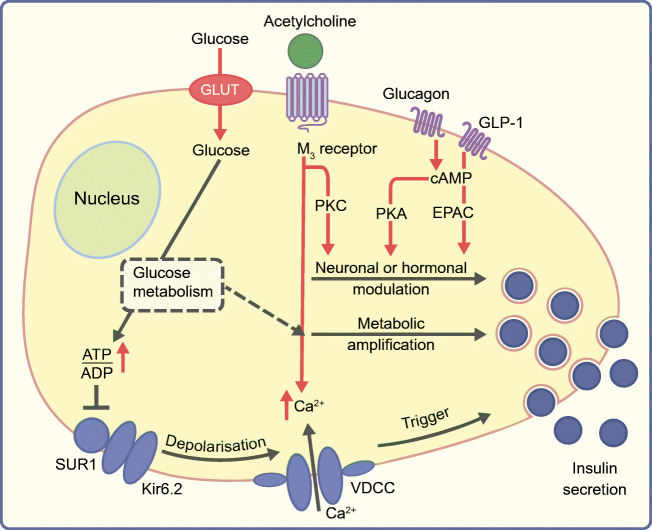
Simplified scheme of stimulus–secretion-coupling pathways in the pancreatic beta cell and possible interaction points for alpha cell-derived paracrine signals. The triggering pathway of GSIS consists of glucose uptake through glucose transporters (GLUT), glucose metabolism, increase in the ATP/ADP ratio, closure of K_ATP_ channels (SUR1/Kir6.2) and opening of voltage-dependent Ca^2+^ channels (VDCC). This Ca^2+^ influx is the trigger for insulin release. The amount of insulin released is then modified by either metabolic amplification or modulation by extracellular inputs involving signalling through GLP-1 or glucagon receptors and cAMP/PKA/EPAC or through acetylcholine receptors and phospholipase C (PLC)/diacylglycerol (DAG)/protein kinase C (PKC) (denoted by PKC). The depicted extracellular inputs and subsequent signalling pathways represent the points of interaction for alpha cell derived paracrine signals. This figure is available as part of a downloadable slideset

Pancreatic beta cells release insulin in response to a rise in plasma glucose concentration. Glucose-stimulated insulin secretion (GSIS) involves multiple pathways: the triggering pathway, the metabolic amplifying pathway and neuronal or hormonal modifying pathways (Fig. 1). In the triggering pathway, ATP (generated by glucose metabolism) and Ca^2+^ influx are the principal signals. Glucose is taken up through the insulin-independent glucose transporter GLUT2 (*SLC2A2*, rodents) or GLUT1 and GLUT3 (*SLC2A1* and *SLC2A3*, humans), phosphorylated by glucokinase and metabolised to produce ATP. The resulting rise in the ATP/ADP ratio leads to the closure of ATP-dependent K^+^ (K_ATP_) channels, membrane depolarisation and the opening of voltage-gated Ca^2+^ channels. The resulting increase in cytosolic free Ca^2+^ ([Ca^2+^]_i_), in turn, triggers exocytosis of insulin granules. The electrical processes involved are reviewed in [6–8]. The signals of the metabolic amplifying pathway as defined by Henquin [9] are still not fully explained, but are thought to include metabolism itself, as well as factors such as the increase in NADPH or ATP and a concurrent decrease in MgADP [10, 11]. In addition, metabolism-derived cAMP generation might contribute to the metabolic amplification [12]. Other important second messengers influencing insulin secretion include phospholipid-derived molecules, such as diacylglycerol, inositol polyphosphates [13] and cAMP (for review, see [12]). Activation of the cAMP/protein kinase A (PKA)/exchange protein directly activated by cAMP (EPAC) system by stimulation of the G-protein-coupled glucagon-like peptide 1 (GLP-1) or glucagon receptors in particular leads to a potentiation of GSIS but not to a triggering of insulin secretion on its own (Fig. 1). Similarly, activation of muscarinic acetylcholine receptors and subsequent initiation of the phospholipase C (PLC)/diacylglycerol (DAG)/protein kinase C (PKC) cascade also improves GSIS (Fig. 1, Cascade simplified as ‘PKC’) [14]. These release-modifying mechanisms are the interaction points for modulation of insulin secretion by innervation, hormonal input or paracrine interactions.

## Factors released by the alpha cell

Like the beta cell, the alpha cell contains and releases, a number of factors besides its major hormonal product, glucagon, that can have both autocrine and paracrine signalling properties [3].

**Fig. 2:**
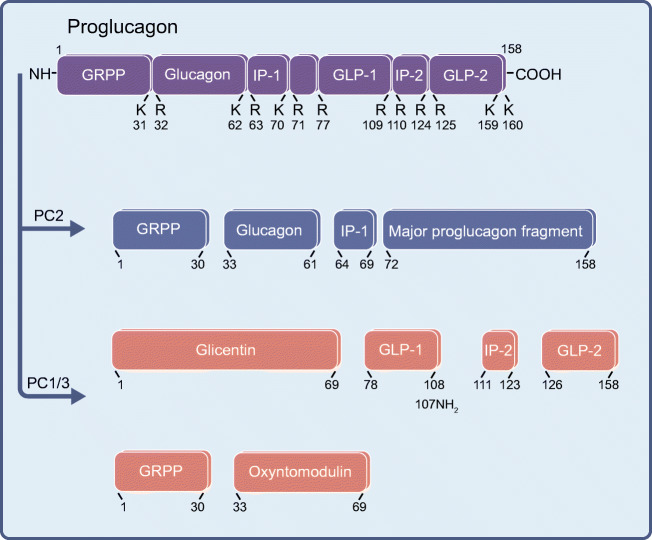
Schematic representation of the post-translational processing of proglucagon. The proglucagon peptide is cleaved by either PC2 to generate glucagon, glicentin-related polypeptide (GRPP), intervening peptide 1 (IP-1) and the major proglucagon fragment or by prohormone convertase 1/3 (PC1/3) to generate GLP-1, GLP-2, glicentin or GRPP, intervening peptide 2 (IP-2) and oxyntomodulin. Pancreatic alpha cells normally express PC2, while L cells of the duodenal duct and neurons express PC1/3. The prohormone convertases cleave proglucagon at specific pairs of the basic amino acids lysine (K) and arginine (R). GLP-1 can be produced in two isoforms: GLP-1(7–37) (corresponding to amino acids 78–108 of the proglucagon peptide) and GLP-1(7–36NH_2_) (78–107NH_2_); the second isoform is generated by α-amidation of the carboxyl-terminal arginine residue. This figure is available as part of a downloadable slideset

The most important product of the alpha cell is the hormone glucagon. Glucagon is expressed from the preproglucagon gene (*GCG*), a gene that is also active in intestinal L cells and in parts of the central nervous system [15]. In the pancreatic alpha cell, proglucagon is processed by prohormone convertase 2 (PC2), resulting in the generation of glucagon and the so-called major proglucagon fragment, while in L cells, proglucagon is processed by PC1/3 resulting in the generation of GLP-1, among other products [16] (Fig. 2). This alternative processing product is an incretin hormone, which is able to potentiate the release of insulin under conditions of elevated blood glucose. Modulation of the incretin signalling system by either GLP-1 analogues directly or by stabilising the circulating GLP-1 by inhibition of dipeptidyl peptidase 4 (DPP4) is currently used as a treatment strategy for type 2 diabetes [17].

Another important product secreted by the alpha cell is glutamate. It is co-released with glucagon and acts through ionotropic glutamate receptors as a positive feedback signal for enhanced glucagon release under low-glucose conditions [3, 18]. These particular α-amino-3-hydroxy-5-methyl-4-isoxazolepropionate (AMPA)/kainate receptors are not expressed in beta cells. However, beta cells express other glutamate receptors, such as *N*-methyl-d-aspartate (NMDA) receptors, but the consequences of glutamate signalling appear to be quite contradictory, with both positive and negative consequences for beta cell function and survival [19]. In addition, glutamate could influence beta cell function by uptake through glutamate transporters and the ensuing depolarising current [6]. Finally, glutamate is also produced by the beta cell itself during glucose metabolism and this endogenous glutamate influences incretin-induced insulin secretion [20]. Glutamate is also the substrate for the synthesis of GABA, which is an important paracrine signal released by the beta cell that inhibits glucagon-release from alpha cells [21, 22].

Finally, at least in humans, alpha cells are able to produce and release acetylcholine, a neurotransmitter normally released by parasympathetic nerve-endings in mouse islets, which functions as a paracrine signal sensitising the beta cell to the prevailing glucose concentration [3, 23].

## Effects of glucagon on the pancreatic beta cell

The stimulatory effect of glucagon on insulin secretion from pancreatic beta cells was established as long ago as 1965 [24] and has since been shown numerous times in a number of different model systems [25–27]. It was demonstrated that isolated sorted beta cells exhibit reduced cAMP content [28] and GSIS [29]. The presence of alpha cells, or of glucagon at least, improves the response of isolated pancreatic beta cells to a glucose stimulus [28–30]. It was also shown that islets with a higher alpha cell content, i.e. islets from different parts of the rat pancreas, show improved GSIS compared with islets with a lower alpha cell content, a difference that could be eliminated by the addition of exogenous glucagon [31]. Mechanistically, the positive effect of glucagon on beta cell function is achieved by glucagon binding to the glucagon receptor, a G-protein-coupled receptor (GPCR) that is expressed in beta cells, and the activation of the cAMP/PKA/EPAC system [3, 12, 26, 27, 32]. The importance of glucagon receptor signalling for beta cell function was confirmed by a positive effect of beta cell specific overexpression of the glucagon receptor [33]. Additionally, glucagon appears to also activate the GLP-1 receptor, which is a GPCR that triggers similar downstream signalling events in the pancreatic beta cell [34]. The activation of the cAMP/PKA system improves GSIS by activating neuronal or hormonal modulatory pathways of insulin secretion (see above), resulting in an increase in the number of vesicle fusion events and in intracellular vesicle transport, with the net result of enhancement of GSIS; however, insulin secretion is not stimulated in the absence of glucose [11, 12]. This makes glucagon a modulatory signal akin to GLP-1 or glucose-dependent insulinotropic peptide (GIP).

## Is glucagon a paracrine signal in vivo?

While glucagon and the presence of alpha cells undoubtedly have a positive effect on GSIS from pancreatic beta cells in vitro, its role as a paracrine factor in rodent islets in vivo has been questioned. Rodent islets consist of an almost exclusive beta cell core surrounded by a mantle that contains the other pancreatic endocrine cell types. The majority of beta cells are neighboured by other beta cells and only a minority are in direct contact with alpha cells [35]. A paracrine effect could also be exerted through the intra-islet vasculature, but it has been proposed that the alpha cells are downstream of the beta cells in the blood flow through the rodent islets [36–38]. In rat islets it was demonstrated that perfusion with an anti-glucagon antibody, which should counteract any glucagon-driven paracrine effect from the alpha cell, had no effect on GSIS, while reverse perfusion with the same antibody, switching the flow from beta cell > alpha cell to alpha cell > beta cell, led to a 30% reduction of GSIS [36–38]. These studies suggested that a paracrine effect of glucagon on pancreatic beta cells in rodents is probably relatively minor. This view of rodent islet blood flow has long been under discussion (see [39] for the different models of islet microcirculation) and new observations utilising in vivo imaging of islet blood flow challenge even these models [40], making the issue of paracrine effects through the bloodstream for rodent islets open for debate. Another study arguing against the importance of a paracrine interaction between alpha and beta cells involved the generation of the glucagon-DTR mouse, a mouse that expressed the diphtheria toxin receptor (DTR) in pancreatic alpha cells. Almost complete ablation (i.e. a 97.4% reduction) of alpha cells by diphtheria toxin injection into adult glucagon-DTR mice had no measurable effect on glucose homeostasis [41]. Apparently, the surviving 2.6% of alpha cells were sufficient for the maintenance of normal glucose homeostasis. Beta cell function was surprisingly unaltered according to glucose tolerance examined in vivo and GSIS assessed in vitro. These findings demonstrate that the intrinsic capabilities of the beta cell were sufficient to maintain normoglycaemia under ‘normal’ circumstances. However, the consequences of beta cell stressors, such as ageing, pregnancy or a diet challenge were not investigated in this mouse model. On the other hand, investigation of insulin secretion in perfused pancreases from wild-type, global glucagon receptor knockout (KO), beta cell-specific glucagon receptor KO and GLP1 receptor KO mice and mice in which alpha cells were ablated in a similar manner as mentioned above [41], demonstrated that glucagon signalling through either glucagon or GLP-1 receptors is able to potentiate GSIS [34]. Another study supporting the notion of intra-islet glucagon signalling as a positive signal for GSIS utilised the alpha cell-specific expression of an inhibitory designer GPCR (GiD) to acutely inhibit glucagon secretion from pancreatic alpha cells [42]. Mice expressing this receptor and treated with the specific GiD activator clozapine *N*-oxide (CNO) displayed an impaired glucose tolerance associated with a lack of GSIS in vivo [42]. The reduction of GSIS during glucagon secretion by inhibition of alpha cell activity was rescued by exogenous glucagon and recapitulated by the inhibition of glucagon- and GLP-1 receptors [42]. It has also been shown that exogenous glucagon or stimulation of alpha cells during hyperglycaemia stimulates insulin secretion, an effect that is dependent on both glucagon and GLP-1-receptors on the beta cell [43, 44].

How important paracrine interactions between alpha cells and beta cells are for the proper functioning of rodent islets has yet to be completely settled. Maybe cAMP generated through innervation or by GLP1 signalling in vivo is sufficient to compensate for the loss of glucagon-secreting alpha cells, something that cannot occur in isolated islets or isolated beta cells.

## Differences in islet structure and their implication for paracrine interaction

**Fig. 3:**
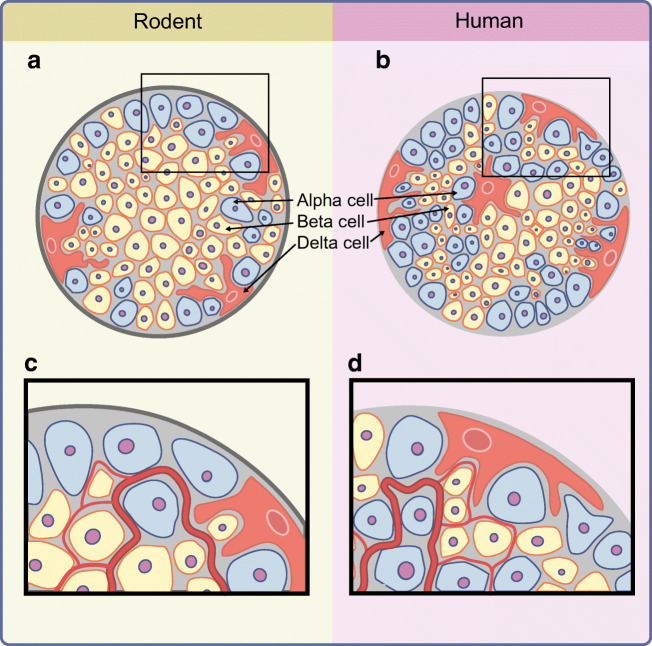
Schematic representation of the structure (**a**, **b**) and vasculature (**c**, **d**) of rodent (**a**, **c**) and human islets (**b**, **d**). Alpha cells are depicted in blue, beta cells in yellow and delta cells in orange. Other endocrine and non-endocrine cell types usually present in the islet have been omitted from this figure for clarity. The islet area, representing islet-specific extracellular matrix proteins, is shown as a grey background, while the particularly strong peri-islet basement membrane of rodent islets is indicated by the bold grey outline. Blood vessels are depicted in red (**c**, **d**). The illustration is based on observations published in [3, 35, 45–51]. This figure is available as part of a downloadable slideset

When comparing rodent islets with human islets, it was demonstrated that the cellular composition of the islets and, almost more importantly, the distribution of the endocrine cells within the islet are different [35, 45–48] (Text box). When looking beyond humans and rodents, the diversity of islet architectures becomes even greater [49]. While a lot of species from groups as diverse as rodents, rabbits, elephants, dogs, seals and toothed whales adhere to the core–mantle paradigm, cats and horses show a reverse core–mantle structure with alpha cells in the centre, and pigs show a more lobular islet structure [35, 49]. In humans and non-human primates the endocrine cells within the islets are intermingled [49]. Islet structures outside mammals are more heterogeneous [49]. Furthermore, it was demonstrated that islet architecture and beta:alpha cell ratio in mice can vary depending on factors like pregnancy, age or different models of diabetes, such as the *ob*/*ob* mouse, which has an extremely high beta cell content, and the *db*/*db* mouse, which as an increased alpha cell content [47, 49, 50]. The variability of islet structures in different species was proposed to be an evolutionary acquired adaptation induced by the physiological conditions of each species rather than just disparities between species without any functional implications [49]. As stated above, rodent islets have a core–mantle structure (Text box, Fig. 3a), but in human islets, alpha and beta cells are intermingled (Text box, Fig. 3b). Furthermore, the alpha cell content is significantly higher than in mouse islets, although the literature documents an apparent high variability [35, 45, 46] (Text box). Nevertheless, a consequence of the different distribution and different proportions of alpha and beta cells is that the majority of beta cells are in direct contact with an alpha cell. While in mouse islets only 28% of beta cells have heterotypic cell–cell contact and 71% have only homotypic contacts, these numbers are reversed in human islets [35] (Text box). This means that the majority of beta cells in human islets are in direct contact with alpha cells. It has also been demonstrated that in human islets at least some beta cells appear to partially envelop alpha cells [45]. Furthermore, because of the random arrangement of alpha and beta cells in human islets, when it comes to the microcirculation within the islet, the beta cells are not always ‘upstream’ of alpha cells [3, 35, 51] (Fig. 3c, d), independent of the model used for blood flow. The differences in cytoarchitecture (Fig. 3a, b) and organisation of beta and alpha cells along the vasculature (Fig. 3c, d) between human and rodent islets have implications for the possibility and the importance of paracrine interactions between alpha and beta cells. Because of this, it is important to study human islets and not completely rely on rodent models for the study of islet physiology and pathophysiology.

## Alpha cells as a source of cholinergic signals in human islets

**Fig. 4:**
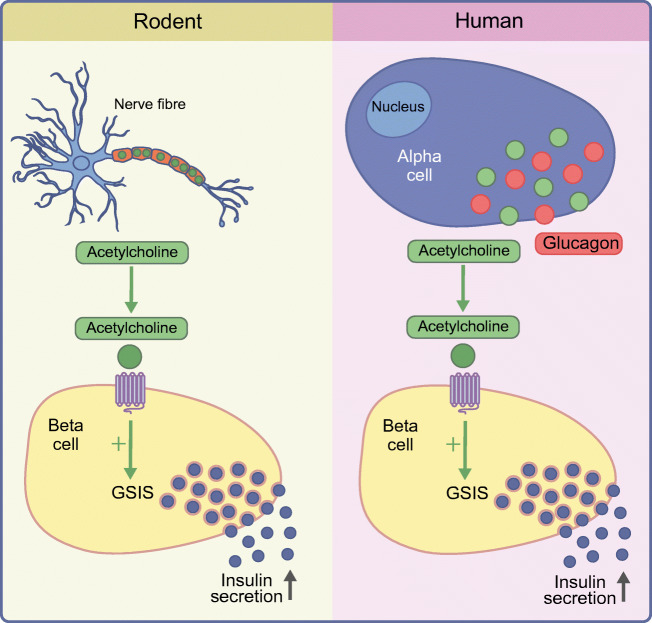
Schematic representation of the difference in cholinergic signalling in mouse and human islets. Mouse islets are richly innervated by parasympathetic nerve fibres and acetylcholine secreted by these fibres contributes to improved GSIS. In human islets, which are not innervated by parasympathetic fibres, acetylcholine is secreted by pancreatic alpha cells. This figure is available as part of a downloadable slideset

Rodent islets are richly innervated by sympathetic and parasympathetic fibres, and signals from the autonomous nervous system play an important role in the maintenance of proper GSIS from pancreatic beta cells [52, 53]. Metabolic transplantation experiments have demonstrated that this cholinergic innervation has a strong influence on GSIS in vivo*.* Metabolic transplantation means the transplantation of a sufficient amount of islets to reverse streptozotocin-induced diabetes, thereby replacing the endogenous islets with islets transplanted either into the anterior chamber of the eye, under the kidney capsule or at a different transplantation site. When islets from C57BL/6J mice were transplanted into the anterior chamber of the eye, it was observed that glycaemia as well as glucose handling after an i.p. glucose tolerance test was improved by ambient light compared with measurements performed in the dark or measurements in mice with islets transplanted under the kidney capsule [54]. This was explained by the cholinergic nervous input from the iris, which was thereby being influenced by the pupillary light reflex. If islets from 129X1 mice were used for the same experiments, the influence of the pupillary light reflex on glucose handling was lost. This is because the islets of these mice have much sparser parasympathetic innervation in the pancreas, which is retained after transplantation [54]. When looking at patterns of parasympathetic innervation in human pancreatic islets it was noted that human islets not only demonstrate weak parasympathetic innervation but that alpha cells were positive for the classical marker of parasympathetic neurons, namely, the vesicular acetylcholine transporter (vAChT) [23]. Human alpha cells were also found to express choline transporter 1 (ChT1) and choline acetyltransferase (ChAT). The presence of these three proteins was also confirmed in isolated, denervated human islets by western blot and mRNA expression analysis, suggesting that human alpha cells are able to produce and secrete acetylcholine [23]. The release of acetylcholine from isolated human islets was confirmed by detecting acetylcholine using biosensor cells [23, 55]. Acetylcholine secretion was increased after stimulation with either kainate or low glucose, classical stimuli for the alpha cell. To confirm the importance of acetylcholine as a paracrine signal, islets were challenged with an experimental protocol that alternately stimulated alpha and beta cells in the presence of modulators of the acetylcholine signalling system. While an M_3_ receptor-specific antagonist reduced insulin responses to repeated stimulation with 11 mmol/l glucose, an acetylcholinesterase blocker led to increased insulin secretion under these conditions [23]. Finally, when human islets were used for metabolic transplantation into the anterior chamber of the eye, the light sensitivity of blood glucose control observed when mouse islets were transplanted into the eye was lost, indicating that cholinergic innervation from the iris is not important for beta cell function in human islets [56]. Taken together, the results demonstrated that, in addition to influencing beta cells through the paracrine effect of glucagon release, human alpha cells modulate beta cell function by releasing acetylcholine, thereby ‘replacing’ the parasympathetic input observed in rodent islets (Text box, Fig. 4).

## Paracrine interaction between alpha and beta cells helps to determine the glycaemic set point

Normoglycaemia is a species-specific value that probably reflects an evolutionary adaptation to the diet and metabolism of different animals. Human normoglycaemia is maximally 6.1 mmol/l, but is usually about 5 mmol/l, while mean normoglycaemia (the glycaemic set point) in C57BL/6J mice is approximately 8.3 mmol/l, in nude mice (used as transplantation recipient), it is 6.1 mmol/l and in the cynomolgus monkey (used as a islet donor in addition to the human and mouse donors), it is 2.8 mmol/l [56]. Xenotransplantation studies showed that the donor normoglycaemia was transferred to the host by the transplantation of islets [56–58]. The lower set point for blood glucose in mice transplanted with human or cynomolgus monkey islets was independent from the islet mass transplanted, but depended on the donor islet species and is therefore likely to be dependent on islet composition [56]. As mentioned above, human and monkey islets have a different cytoarchitecture from the mouse islet, allowing a more comprehensive contact between alpha and beta cells and more paracrine interactions [35, 45, 46]. By injecting mice transplanted with human islets with an antagonist against the human glucagon receptor, the blood glucose level in these animals was changed from ‘human’ blood glucose to the blood glucose observed in nude mice, suggesting that the glucagon signal from human alpha cells is indeed responsible for determining the glucose set point [56]. Interestingly, islets from 129X1/SvJ mice also conferred a lower glycaemic set point than islets from C57BL/6J mice and were shown to contain more alpha cells than C57BL/6J islets [56]. This suggests that it is not necessarily the species difference, but the different alpha:beta cell ratio that is the critical variable. Paracrine interactions between alpha and beta cells therefore appear to be the determining factor for the set point of glucose homeostasis in the organism. It remains to be investigated how the different islet architectures observed in different species correlate with their glycaemic set points.

## Are alpha cells a possible source of GLP-1?

As mentioned above, pancreatic alpha cells normally express PC2, which results in the processing of proglucagon to glucagon. Intestinal L cells that release GLP-1 express PC1/3 and consequently process proglucagon to GLP-1 (Fig. 2). Whether alpha cells also produce and release GLP-1 has been investigated in various systems. Expression of PC1/3 and production of GLP-1 in pancreatic alpha cells has been observed after treatment with IL-6 [59], during regeneration of beta cell mass after streptozotocin treatment in neonatal rats [60], during the development of a type 2 diabetes-like phenotype in the gerbil *Psammomys obesus* [61] or pregnancy in mice [62]. It was also demonstrated that at least a subset of alpha cells in human islets express PC1/3 and are therefore producing and releasing GLP-1 in response to physiological stimuli [63]. Both human and mouse islets produce the biologically active GLP-1(7–37) [34, 63, 64] and GLP-1(7–36NH_2_) has been detected in human islets [63]. It is noteworthy, however, that in normal mice, GLP-1 release in perfused pancreas experiments is low, at or below the detection limit of the assay [34]. On the other hand, these measurements say very little about the concentration of GLP-1 within the islet. This local GLP-1 signalling system appears to be activated during the development of type 2 diabetes and beta cell stress [65–67]. The ‘reprogramming’ of at least a larger subset of alpha cells to produce GLP-1 instead of glucagon might be beneficial in the treatment of type 2 diabetes since GLP-1 is not only more efficient in enhancing GSIS, but it also does not affect hepatic glucose output.

## How to further investigate alpha cell–beta cell interactions in human islets

**Fig. 5:**
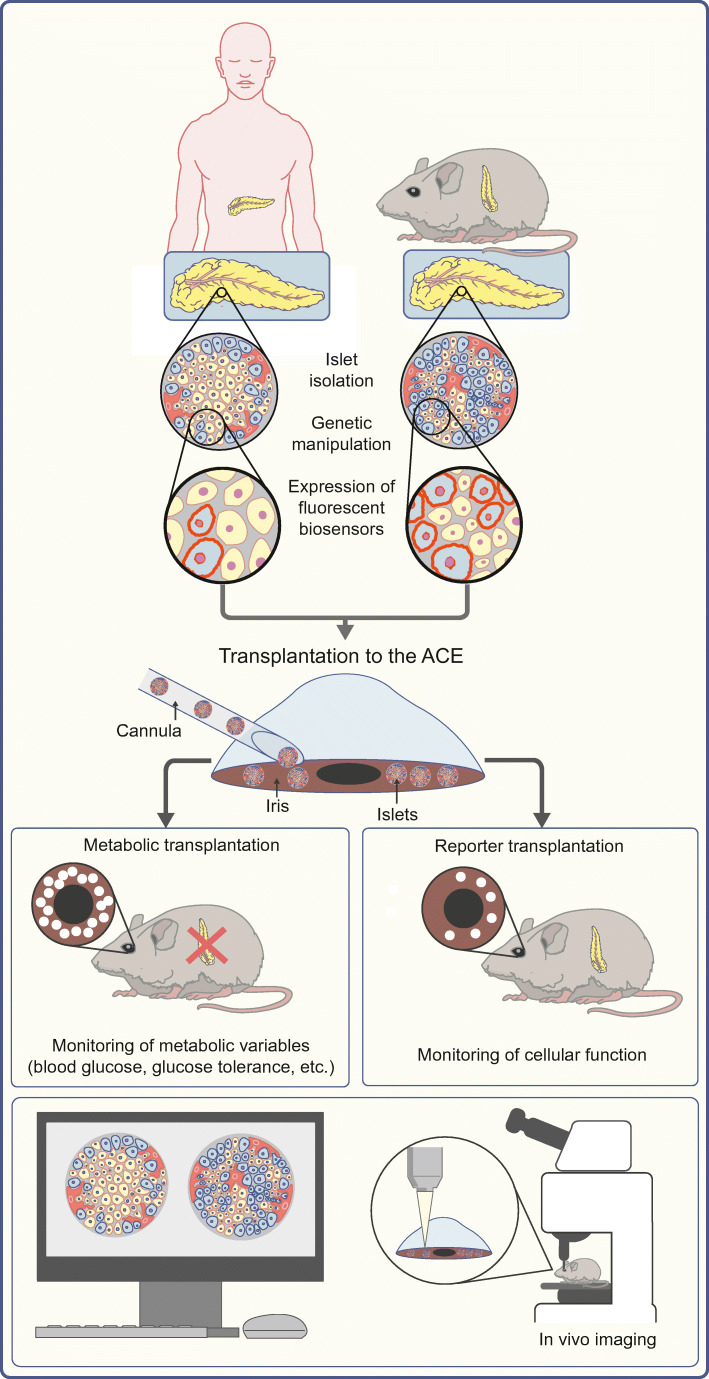
Islet transplantation into the anterior chamber of the eye (ACE) as a tool to study human islet physiology/pathology in vivo. Islets from either mouse or human donors are isolated, then equipped with genetically encoded fluorescent biosensors and/or modified by CRISPR/cas9 genome editing and transplanted to the anterior chamber of the eye of immunocompromised mice (represented as white dots). Metabolic transplantation into streptozotocin-treated recipient mice allows the investigation of the physiological consequences of manipulation of alpha cell–beta cell interactions. Transplantation of a few islets allows the monitoring of alpha and/or beta cell function and survival and alpha cell–beta cell interactions under in vivo-like conditions. This figure is available as part of a downloadable slideset

As we have seen in the past, relying entirely on rodent models to investigate human islet biology and causes and treatment strategies for human diabetes is associated with potential flaws. These are mainly due to the structural and functional differences manifested among various species, which affect paracrine interactions within the islet. However, focusing only on in vitro investigations of human islet function clearly neglects the importance of both the complex interactions in islet biology in vivo and the specific crosstalk with organs in the rest of the body, i.e. nervous system, vasculature, signalling from gastric peptides and the metabolic status of the whole organisms. These factors cannot be fully simulated in an in vitro environment. One way forward might be in the utilisation of a ‘humanised’ mouse model, whereby human islets transplanted into streptozotocin-treated immunocompromised mice are used as a model for human islet physiology/pathophysiology under in vivo conditions [68] (Fig. 5). Transplantation of human islets to the anterior chamber of the eye allows direct observation of various variables of islet morphology and function [69, 70]. Furthermore, the exposed location of the islets in the eye allows for manipulation by local treatment or, for that matter, light, and can illustrate, for example, the effects of parasympathetic innervation [23, 54]. In addition, non-human primates can be used as both donors and acceptors for islet transplantation to the anterior chamber of the eye to study non-rodent islet physiology in vivo [71]. In vivo imaging of islet vasculature [71–73] and islet function [69, 70, 74] have been performed successfully in anaesthetised animals, specifically, the mouse [69, 70, 72–74] and non-human primate [71]. While anaesthesia appeared to be unproblematic for studies of islet vasculature, there was an inhibitory effect of some anaesthetics on GSIS without affecting beta cell Ca^2+^ handling [74]. The potential effects of various anaesthetic agents need to be taken into account when interpreting the data generated by in vivo imaging experiments.

Combining the in vivo imaging approach with the expression of fluorescent biosensors for different variables of beta and/or alpha cell function and survival, as well as adding genetic manipulation of human islet cells using the CRISPR/cas9 system, will lead to a better understanding of human islet cell biology/pathology and specifically the role of alpha cell-derived paracrine signals for proper beta cell function and survival.

## Electronic supplementary material

Slideset of figures (PPTX 768 kb)

## Abbreviations

cAMP: Cyclic adenosine monophosphate
EPAC: Exchange protein directly activated by cAMP
GABA: γ-Aminobutyric acid
GiD: Inhibitory designer GPCR
GLP-1: Glucagon-like peptide 1
GPCR: G-protein-coupled receptor
GSIS: Glucose-stimulated insulin secretion
K_ATP_: ATP-dependent K^+^ (channel)
PC: Prohormone convertase
PKA: Protein kinase A
