# Supplementary material for: Alpha cell regulation of beta cell function
Source: Diabetologia. 2020 Sep 7;63(10):2064–75. doi: 10.1007/s00125-020-05196-3 (PMC7476996; doi:10.1007/s00125-020-05196-3)
Supplement: Supplementary file 1 — (PPTX 768 kb) [file 125_2020_5196_MOESM1_ESM.pptx]

## Slide 1
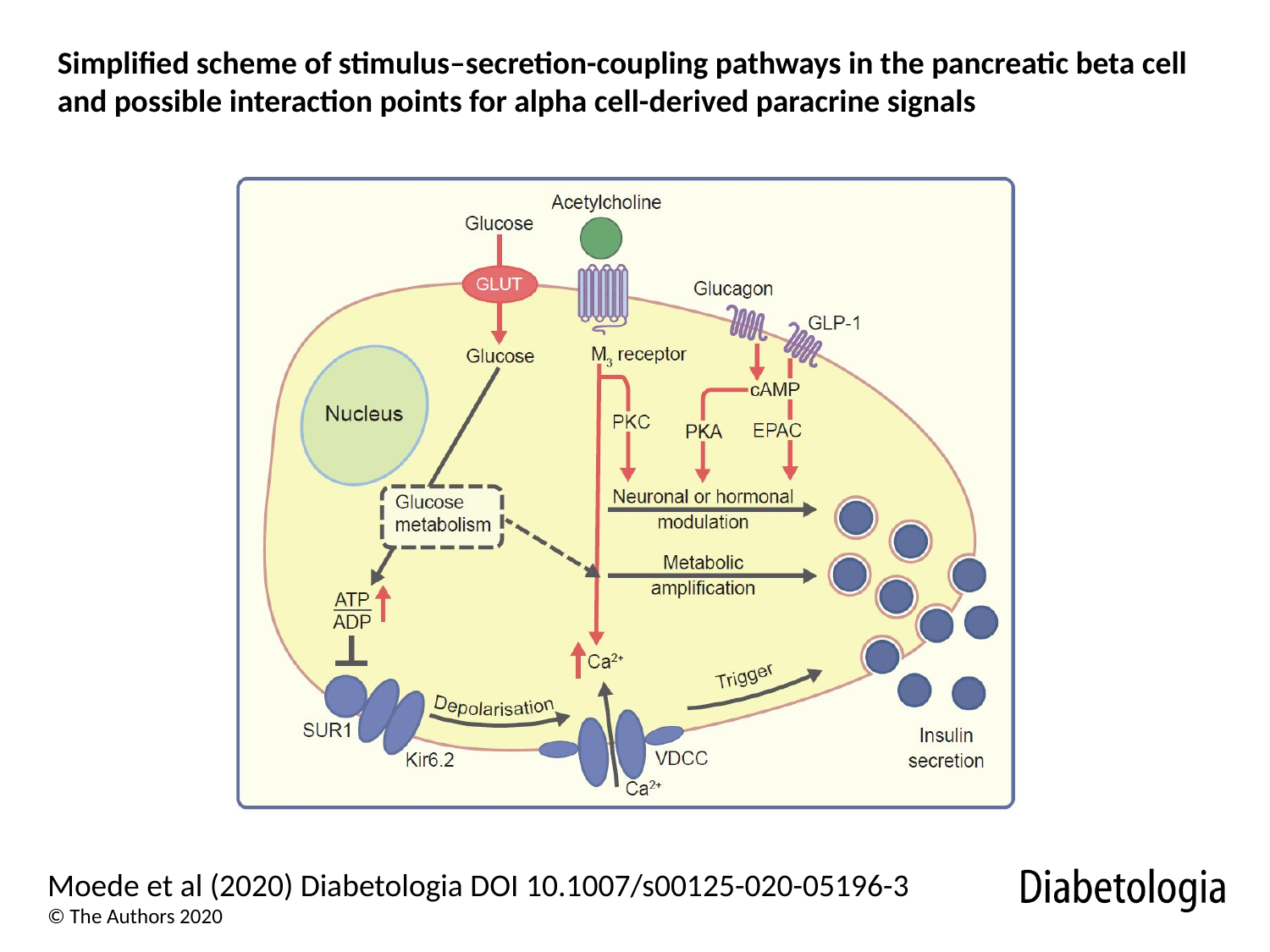

Simplified scheme of stimulus–secretion-coupling pathways in the pancreatic beta cell and possible interaction points for alpha cell-derived paracrine signals
Moede et al (2020) Diabetologia DOI 10.1007/s00125-020-05196-3
© The Authors 2020

## Slide 2
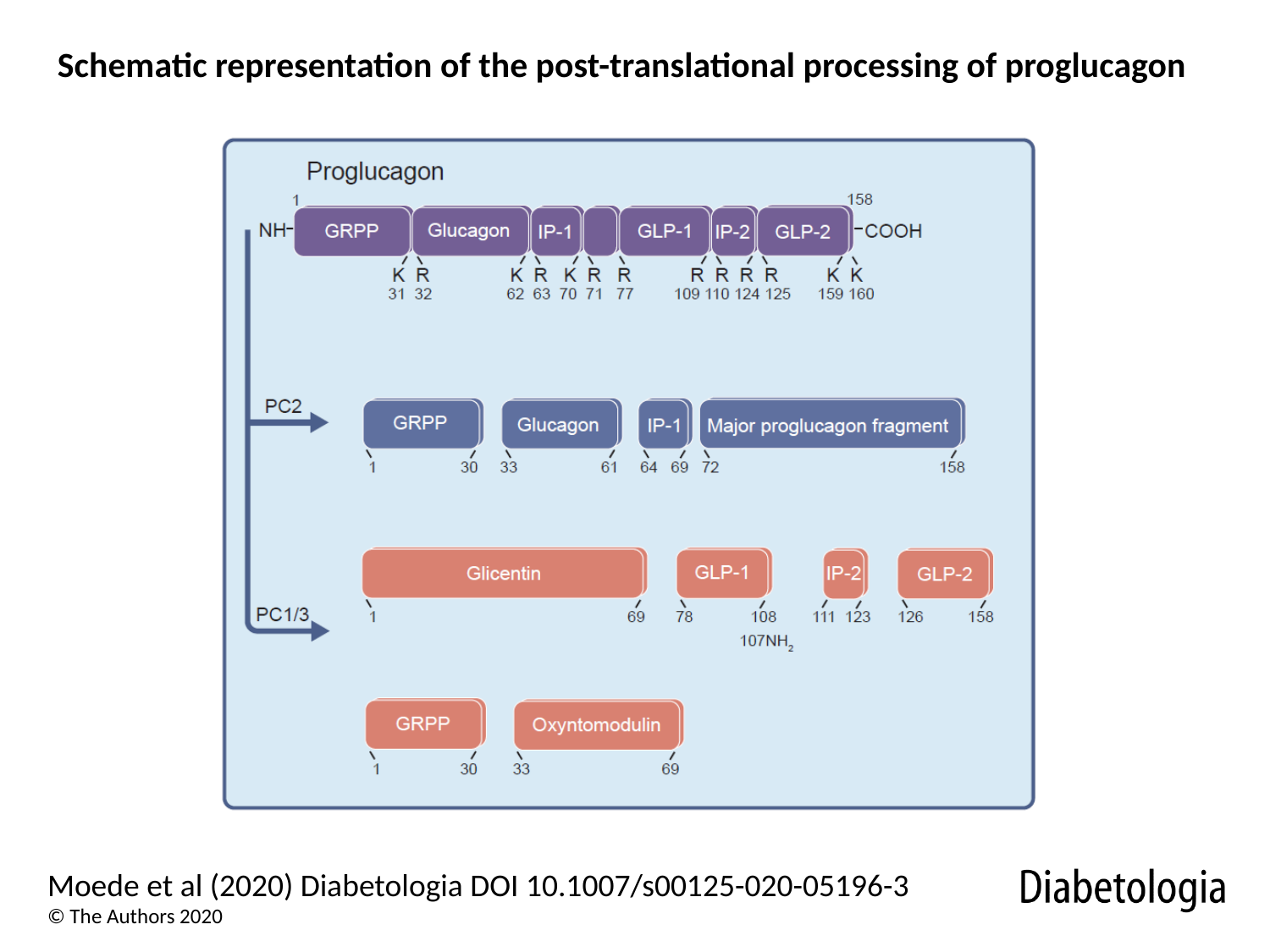

Schematic representation of the post-translational processing of proglucagon
Moede et al (2020) Diabetologia DOI 10.1007/s00125-020-05196-3
© The Authors 2020

## Slide 3
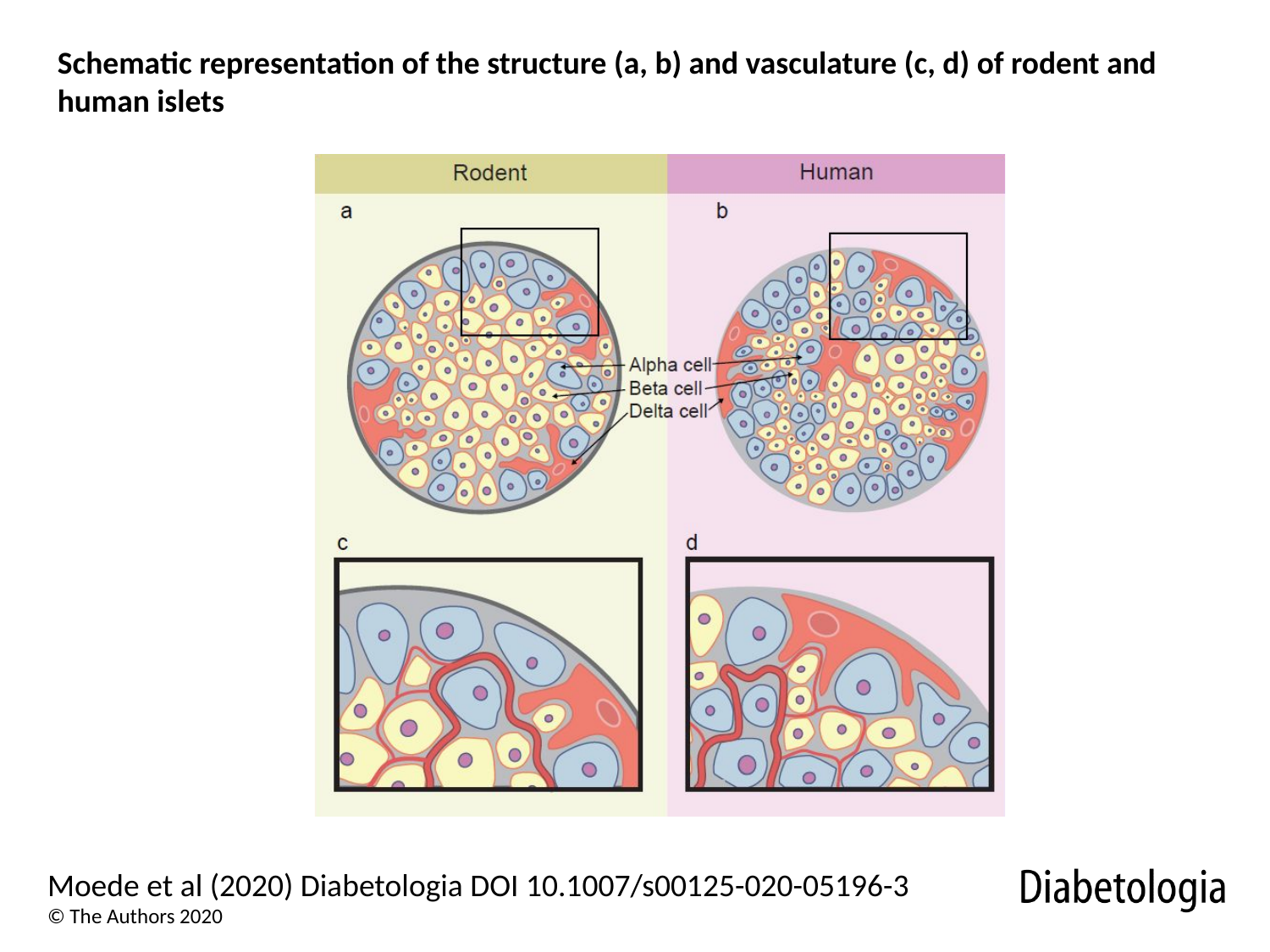

Schematic representation of the structure (a, b) and vasculature (c, d) of rodent and human islets
Moede et al (2020) Diabetologia DOI 10.1007/s00125-020-05196-3
© The Authors 2020

## Slide 4
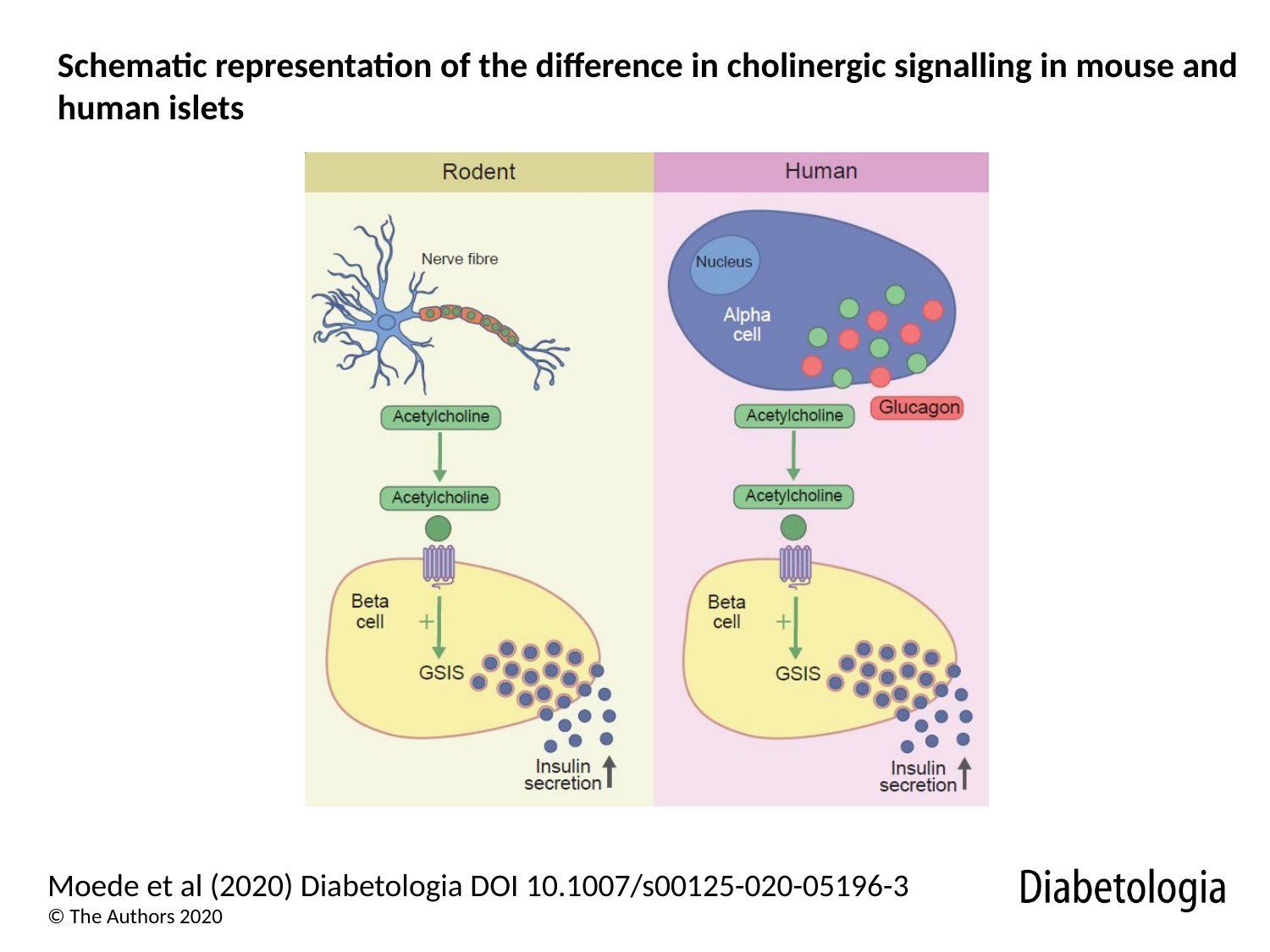

Schematic representation of the difference in cholinergic signalling in mouse and human islets
Moede et al (2020) Diabetologia DOI 10.1007/s00125-020-05196-3
© The Authors 2020

## Slide 5
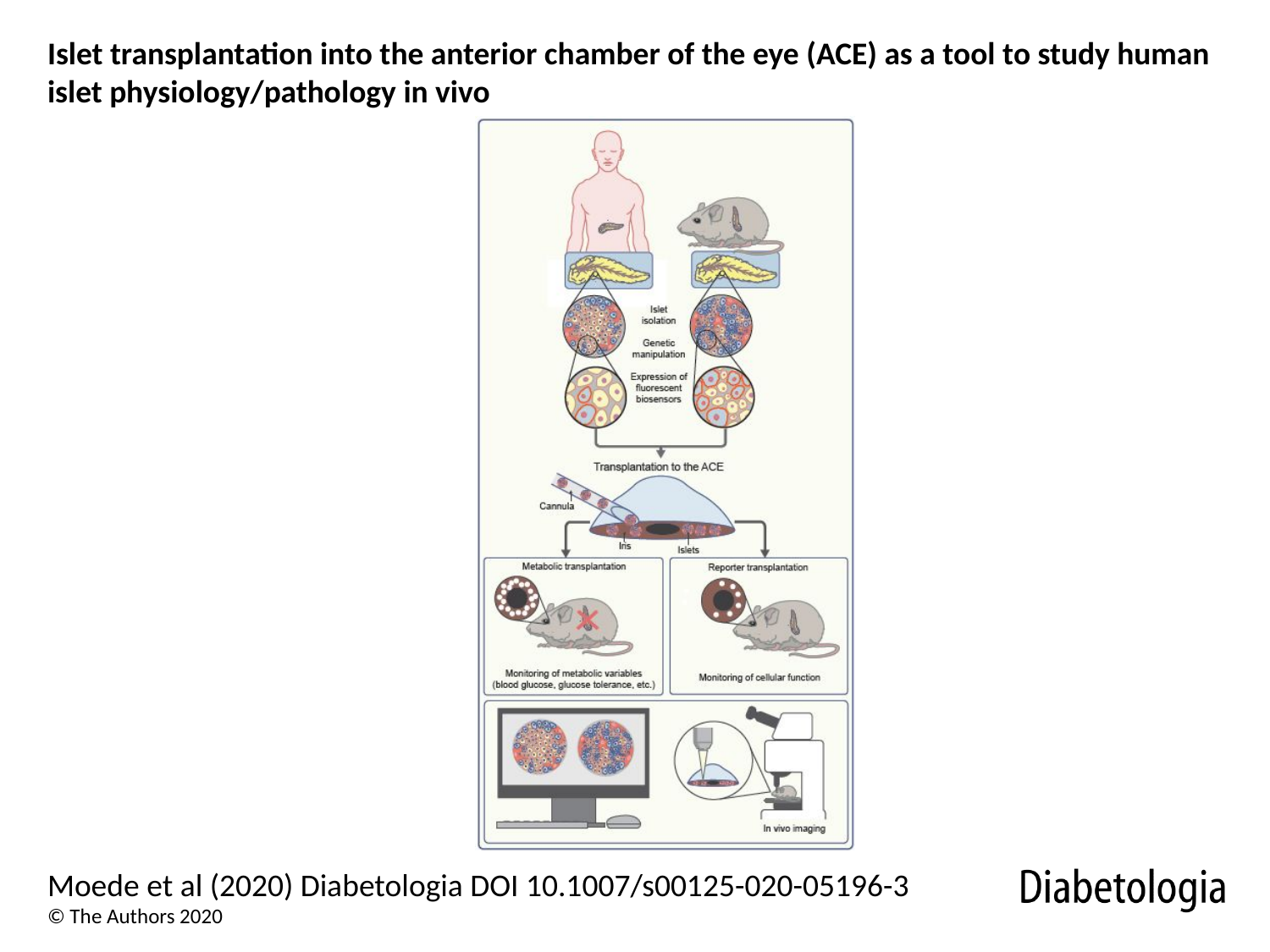

Islet transplantation into the anterior chamber of the eye (ACE) as a tool to study human islet physiology/pathology in vivo
Moede et al (2020) Diabetologia DOI 10.1007/s00125-020-05196-3
© The Authors 2020
